# Type 3 hybrid effectiveness-implementation study implementing and evaluating the Comprehensive High-dose Aphasia Treatment (CHAT) programme in Australian rehabilitation services: a protocol

**DOI:** 10.1136/bmjopen-2026-117575

**Published:** 2026-05-18

**Authors:** Marie-Pier McSween, Jade K Dignam, Kirstine Shrubsole, Rachel Levine, Emma Power, Natasha A Lannin, Sarah J Wallace, Dominique A Cadilhac, Monique F Kilkenny, Annie J Hill, Brooke J Ryan, David A Copland

**Affiliations:** 1 Queensland Aphasia Research Centre, School of Health and Rehabilitation Sciences, The University of Queensland, Brisbane, Queensland, Australia; 2 Surgical Treatment and Rehabilitation Service (STARS) Education and Research Alliance, The University of Queensland and Metro North Health, Brisbane, Queensland, Australia; 3 School of Health and Human Sciences, Southern Cross University, Lismore, New South Wales, Australia; 4 Graduate School of Health, Speech Pathology, University of Technology Sydney, Sydney, New South Wales, Australia; 5 Department of Neuroscience, Monash University, Melbourne, Victoria, Australia; 6 Alfred Health, Melbourne, Victoria, Australia; 7 Stroke and Ageing Research, Department of Medicine, School of Clinical Sciences at Monash Health, Monash University, Melbourne, Victoria, Australia; 8 Stroke theme, Florey Institute of Neuroscience and Mental Health, The University of Melbourne, Melbourne, Victoria, Australia; 9 Centre for Research Excellence in Aphasia Rehabilitation and Recovery, La Trobe University, Melbourne, Victoria, Australia; 10 Curtin School of Allied Health, Curtin University, Perth, Western Australia, Australia

**Keywords:** Speech pathology, Stroke, REHABILITATION MEDICINE, Implementation Science

## Abstract

**Introduction:**

The Comprehensive High-dose Aphasia Treatment (CHAT) programme is an intensive comprehensive aphasia programme, which aims to address evidence-practice gaps in aphasia rehabilitation where there are known barriers to service delivery requiring implementation strategies. The aims of this study are to (1) evaluate the clinical implementation of the CHAT programme, (2) assess the clinical effectiveness of CHAT compared with usual care in rehabilitation services and (3) determine whether the real-world implementation of CHAT compared with usual care is cost-effective.

**Methods and analysis:**

Four participant groups will be recruited across six hospital and health services Australia-wide to participate in a type 3 hybrid effectiveness-implementation study: (1) people with aphasia, (2) support persons, (3) treating clinicians and students and (4) clinical stakeholders (eg, managers). This before-and-after study will include three time periods: (1) ‘usual care’ where people with aphasia will receive their usual care aphasia therapy, (2) ‘implementation transition’ where clinicians will be trained to deliver CHAT and (3) ‘intervention implementation’ where people with aphasia will receive the CHAT programme (ie, 50 hours of evidence-based aphasia therapy over 8 weeks). Evidence-based implementation strategies will be used to facilitate implementation within participating rehabilitation services. The primary outcome is delivery of evidence-based aphasia treatment (ie, CHAT) as measured by a composite score of quality indicators. Clinical effectiveness outcomes, measuring change in language impairment, communication effectiveness, confidence and quality of life, and implementation outcomes will also be examined. We will also conduct an embedded mixed-methods process evaluation and economic evaluation.

**Ethics and dissemination:**

This study has been approved by the Royal Brisbane and Women’s Hospital Human Research Ethics Committee (HREC/2021/QRBW/72154). Outputs will include conference presentations, publications and a training package to optimise implementation of aphasia treatment in rehabilitation service contexts.

**Registration details:**

Australian New Zealand Clinical Trials Registry (ANZCTR) prospective registration ACTRN12621001765819. Trial registered 23 December 2021. https://anzctr.org.au/Trial/Registration/TrialReview.aspx?id=381365&isReview=true.

**STRENGTHS AND LIMITATIONS OF THIS STUDY**

- A hybrid implementation-effectiveness study design will support a rapid research translation and uptake of the Comprehensive High-dose Aphasia Treatment (CHAT) programme at trial completion by identifying effective implementation strategies that can be tailored to local barriers, and provide concurrent answers about intervention effectiveness in real-world settings.
- This study will be conducted across six Australian hospitals and health services, to compare the effectiveness and cost-effectiveness of usual care with the CHAT programme, which has been developed to address known aphasia rehabilitation evidence-practice gaps.
- A diversity of health services will be involved in this study (ie, metropolitan, regional, hospital-based and community-based services), which will allow us to examine implementation in different settings.
- This study only includes people with post-stroke aphasia who speak English, therefore limiting generalisability to other aetiologies and patients from other linguistic backgrounds.
- Due to the non-randomised, pre-post design with the allocation of people with aphasia based on time periods, various risks of bias are possible, including selection bias.

## Introduction

 Aphasia is a language and communication disability that affects approximately 30% of stroke patients,^1 2^ and negatively impacts quality of life more than cancer and Alzheimer’s disease.^3^ Aphasia can have devastating impacts on psychological, social and vocational aspects of life. People with post-stroke aphasia are twice as likely to have depression compared with stroke survivors without aphasia,^4^,^6^ and the presence of aphasia is associated with differences in care quality.^2^ There is high-quality evidence that aphasia therapy improves communication outcomes.^7^,^9^ Furthermore, high-level evidence supports comprehensive, tailored, high-dose aphasia therapy.^9 10^ However, this research is not often translated to clinical practice, leading to evidence-practice gaps.^11^ There are key gaps in therapy timing, intensity, goal setting and communication partner training.^11^,^14^ Addressing these gaps is important for improving aphasia care and patient outcomes.

A solution is the introduction of Intensive Comprehensive Aphasia Programs (ICAPs), including its modified versions.^15^,^19^ ICAPs have been defined as comprising a minimum of 3 hours of daily treatment over a period of at least 2 weeks and have a set start and end date, with a cohort of participants starting and finishing the programme together. ICAPs include individual and group therapy, targeting both impairment and activity/participation levels of language and communication, and include education for patients and/or families.^20^ Modified ICAPs (mICAPs) meet each of these programme elements apart from one element.^21^ There is evidence that ICAPs and their modified versions, such as the Comprehensive High-dose Aphasia Treatment (CHAT) programme,^22^ are effective and can significantly reduce aphasia severity and language impairment, and improve functional communication, psychosocial well-being and quality of life.^1922^,^26^ To increase access to quality aphasia services and to improve the health and quality of life of people living with post-stroke aphasia, the next stage of the research process is to implement mICAPs/ICAPs into clinical rehabilitation services and to evaluate clinical effectiveness, implementation strategy effectiveness and cost-effectiveness.

Successful translation of research into clinical practice takes considerable time and effort^27^ and is more likely if the implementation strategy is tailored to knowledge of barriers and enablers to change.^28^ Trebilcock *et al*^29^ identified barriers to global implementation of intensive aphasia services, with Shrubsole *et al*^30^ more recently investigating barriers to a previous iteration of CHAT (ie, the Aphasia Language, Impairment and Functional Therapy (Aphasia LIFT) programme), highlighting issues like service inflexibility and feasibility concerns within current healthcare constraints. These barriers shaped a tailored implementation strategy for the CHAT programme, incorporating evidence-based behaviour change techniques.^31 32^ The resultant implementation strategy incorporates six components including (1) executive, leadership and stakeholder support; (2) dedicated allocation of clinical staff; (3) interactive education and training programme; (4) resource procurement and provision; (5) ongoing implementation support and (6) consumer engagement and promotion, and is described in more detail elsewhere.^30^ More recently, Monnelly *et al*^33^ also explored the views of 227 speech pathologists regarding intensive and comprehensive aphasia therapy. Barriers raised included a lack of resources for ICAP delivery and a low level of optimism that ICAP delivery issues (eg, insufficient staffing, lack of funder/commissioner support) could be resolved, with only 16.5% of clinicians feeling that their service could be redesigned to implement and deliver an ICAP. This highlights the need for further implementation efforts to enable successful translation of research/clinical recommendations into clinical practice.

The aims of this study are to (1) evaluate the clinical implementation of the CHAT programme, (2) assess the clinical effectiveness of CHAT compared with usual care in rehabilitation services and (3) determine whether the real-world implementation of CHAT compared with usual care is cost-effective. We hypothesise that compared with providing usual care, implementing the CHAT programme using an evidence-based tailored implementation strategy will result in increased quality of care at a service level, as quantified using a composite outcome measure.

## Methods and analysis

### Design and setting

We will undertake a multicentre, pragmatic, prospective, non-randomised before-and-after study with three study periods (usual care, intervention transition, intervention) and an embedded mixed-methods process evaluation and economic evaluation. A type 3 hybrid implementation-effectiveness design will be employed.^34^ A before-and-after study design was selected over a randomised controlled trial due to requirements of the funding model, and to enable the equitable contribution and participation of partner sites. This study will take place across six hospital and health services, across three Australian states. Sites from a wide range of health service settings will take part in this study (ie, metropolitan, regional, in-patient and out-patient hospital-based and community-based services). The diversity of health services involved in this study will allow us to examine implementation in different settings and to enable us to develop a strong plan for future expansion to more organisations that may seek to implement the CHAT programme within Australia.

### Study periods

**Figure 1:**
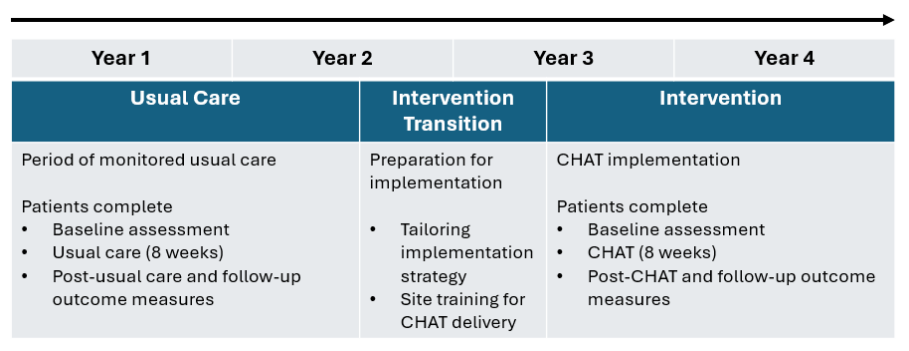
Pragmatic, prospective, non-randomised, before-and-after study design. CHAT, Comprehensive High-dose Aphasia Treatment.

This study will include three time periods (see figure 1): (1) **usual care** (years 1 and 2); (2) **implementation transition** (between years 2 and 3) to allow clinicians to upskill in the implementation and delivery of the CHAT programme; and (3) an **intervention** period (years 3 and 4) for the implementation of the CHAT programme.

#### Period 1: usual care

This first period of the study will examine current speech pathology clinical practices in post-stroke aphasia rehabilitation as prescribed by treating speech pathologists in accordance with health service and site procedures. Patients recruited during the usual care phase will receive standard speech pathology care, as typically provided by the health service. During this usual care period, it will be important for rehabilitation services to avoid, where possible, significant changes to their model of care and service delivery that relate to the CHAT programme. Services will be informed of this requirement and invited to discuss with the research team potential changes that could affect the study. Unavoidable changes will be recorded and monitored by the research team.

#### Period 2: intervention transition

The second period of the study will involve the transition in preparation for the implementation of the CHAT programme. This period will include stakeholders, clinicians and students in the same clinical settings which participated in period 1. During the intervention transition, meetings will be conducted with stakeholders and clinicians at each site to identify potential barriers and facilitators to CHAT implementation, identify site champions and tailor the site implementation strategy, as required. Clinicians and students will be upskilled and trained in accordance with the implementation strategy to deliver the CHAT programme to eligible patients with post-stroke aphasia.

#### Period 3: intervention

##### Comprehensive High-dose Aphasia Treatment programme intervention

Patients recruited during the intervention phase will receive the CHAT programme. The CHAT programme (previously known as the Aphasia LIFT programme in its initial iterations) was first developed and trialled by Rodriguez *et al*,^35^ and most recently, has been successfully implemented in a single metropolitan health service.^22 26^ The programme aims to address known aphasia rehabilitation evidence-practice gaps. This comprehensive aphasia treatment package uses two complementary but often isolated approaches, that is, treatment of the language impairment and its consequences on everyday communication.^35^ The CHAT programme is a modified ICAP, meeting all but the criteria for treatment intensity with respect to the defining ICAP elements. CHAT comprises 50 hours of therapy delivered in a distributed schedule over 8 weeks (see table 1). The programme includes evidence-based and goal-directed individual impairment, activity and participation, and computer-based therapy, and group sessions for education and information sharing, conversational training and discussion of topics related to living with aphasia. The CHAT programme is delivered in cohorts, whereby patients start and complete the programme at the same time. As part of this study, intervention will be delivered in-person with the option of delivery via telerehabilitation to increase access to therapy for regional patients and those discharged from hospital.

**Table 1:** Core components of the CHAT programme

Programme component	No. of sessions
Goal setting session	1 (1–2 hours)
Impairment therapy (*eg, Semantic Feature Analysis, Constraint-Induced Aphasia Therapy*)	14 × 1-hour sessions
Activity and participation therapy—improving communication in context (*eg, Aphasia scripts, role-play, conversational coaching, use of compensatory strategies, conversation partner training*)	14 × 1-hour sessions
Computer therapy (*eg, Aphasia Scripts, StepByStep programme, Constant Therapy*)	14 × 1-hour sessions
Group therapy (*eg, educational sessions on stroke and aphasia, mood and well-being, psychosocial support, living successfully with aphasia*)	8 × 1-hour sessions
Independent practice (home, hospital and community rehabilitation)	–

CHAT, Comprehensive High-dose Aphasia Treatment.

Prior to commencement, individual goal setting in collaboration with the patient will be used to develop an evidence-based plan for treatment across each therapy component, enabling tailoring of the programme to meet patient needs (eg, returning to work, communicating with family members). Treatment planning will occur in collaboration with the treating clinician and the research team.^36^ CHAT seeks to provide participants with the tools to practice language themselves by using commercially available computerised aphasia programmes during treatment. Mobile technology, such as iPads and iPhones, is often used to maintain practice schedules. In addition, the CHAT programme links participants into community resources (eg, peer-led aphasia groups, stroke groups, interest groups and other professionals) to promote psychosocial well-being and participation.

Involvement of partners and family is integral to the programme. Support people, including family members, significant others and/or carers, are invited to participate in all programme elements, as able. This may include attendance at educational group sessions, participation in individual therapy, and technology training and support for computer-based therapy. Depending on the participant with aphasia’s therapy goals, support people may also be the direct recipient of intervention (eg, communication partner training).

##### Implementation strategy

The implementation strategy consists of six components based on behaviour change theories^31 37^ and factors influencing implementation of mICAPs/ICAPs,^11 29 30^ and will be tailored to each setting based on local barrier assessments. The components include the following:

1. **Executive, leadership and stakeholder support:** formal agreements to implement CHAT will be signed with participating health services and pre-implementation discussions will be undertaken with key stakeholders from each site to assess local implementation needs. Multidisciplinary team meetings will be conducted to educate about CHAT and promote the programme.
2. **Specialist web-based educational training:** participating clinicians will complete a web-based educational training component following a site-specific delivery timetable. Training will include background evidence and rationale, the importance of improving aphasia practice, information on CHAT therapy components, treatment techniques and case examples. Video testimonials of people with lived experience of aphasia and the CHAT programme will be included.
3. **Interactive workshop:** after completing the web-based training, clinical teams will attend in-person or online interactive workshops, with a focus on skills development, local barrier identification, problem-solving and team strategy identification.
4. **Resource provision:** essential physical resources will be provided to support implementation, including therapy protocols, goal setting and therapy resources, and digital equipment (eg, iPads).
5. **Ongoing implementation support:** the research team will provide ongoing implementation support to facilitate further barrier identification and collaborative problem solving. Members of the research team will also engage in joint clinical planning with participating clinical teams to monitor team progress and support selection of evidence-based therapies to address goals.
6. **Site champions:** each health service will designate one to two site champions who will monitor and support implementation, identify ongoing barriers and act as intermediaries between the health service and the research team. Site champions will also attend an online monthly community of practice meeting that will focus on promoting peer-learning and sharing implementation experiences across services.

### Treatment of concomitant conditions alongside periods 1 and 3

Relevant concomitant care and interventions will continue alongside this study. Examples of other interventions that are permitted during the study include other areas of speech pathology practice (eg, swallowing, motor speech, voice, augmentative and alternative communication, fluency) as indicated, as well as other allied health and medical care/intervention. Participants will not be prohibited from accessing non-aphasia therapy-related services during their participation in this study.

#### Participants and recruitment

This study will involve four participant groups: (1) people with post-stroke aphasia, (2) their support person, (3) treating clinicians and students and (4) non-treating clinical stakeholders (eg, directors of speech pathology). Consent to participate in this study will be obtained from all participants following the provision of a written participant information and consent form (with an aphasia-friendly version for people with aphasia, see online supplemental file 1) and a face-to-face or online meeting to provide verbal explanation and further discuss participation in this research.

#### People with aphasia

People with post-stroke aphasia will be recruited from participating sites if they are aged 18 years or older at the time of consent, have had a documented stroke and present with aphasia as identified by a speech pathologist using either the Language Screening Test^38^ or the Comprehensive Aphasia Test (CAT^39^). People with aphasia who have significant comorbidities that would prevent participation in aphasia rehabilitation (as determined by the treating speech pathologist in consultation with the multidisciplinary team and research team) or who did not have functional English language skills prior to their stroke will be excluded. Due to the between-group design of this study, people with aphasia who have previously been enrolled in period 1 of the study (ie, usual care) will not be eligible to participate in period 3 of the study (ie, intervention). Eligible people with aphasia will be identified by site champions and speech pathologists within the service, in consultation with the treating medical team and the research team.

#### Support people

Support people (ie, family members, significant others and/or carers of eligible and consented people with post-stroke aphasia) will be recruited and consented to participate in this study. Support people will be nominated by people with aphasia and confirmed as potential participants for the study by site champions and speech pathologists within the service in consultation with the treating medical team and the research team. There are no formal inclusion criteria for support people; however, they should play a key role in the person with aphasia’s life and rehabilitation journey. Inclusion of a support person is not a requirement for patients to receive CHAT.

#### Treating clinicians and students

Qualified speech pathologists and allied health assistants employed by the health service who are working in aphasia rehabilitation and delivering treatment as part of the usual care and/or CHAT programme will be eligible to participate. All other relevant staff involved in the delivery of the CHAT programme within their clinical setting (eg, social workers, psychologists, occupational therapists) will also be eligible to participate. Students on placement at participating sites may also be involved and considered eligible. All key treating clinicians and students will be identified based on caseload allocation and invited to participate by principal investigators, site champions or members of the research team prior to and during periods 1, 2 and 3.

#### Non-treating stakeholders

Stakeholders supporting the implementation of the programme within their clinical setting but not involved in the delivery of usual care and/or the CHAT programme will be eligible to participate. This includes speech pathologists and allied health assistants not involved in delivery of usual care and/or the CHAT programme, allied health professionals, nursing staff, administrative and support staff, medical staff, and service/clinical managers supporting the implementation of the CHAT programme at participating sites. All key non-treating stakeholders will be identified and invited to participate in the project by principal investigators, site champions or members of the research team prior to and during periods 1, 2 and 3.

## Outcomes

### Implementation effectiveness

The primary outcome of this study is the proportion of high-quality comprehensive aphasia treatment received, which will be quantified in a composite measure (see tables 2 and 3). This measure of treatment quality is based on research evidence^40^ and stakeholder experience and preferences.^41 42^ Each component (ie, clinical quality of aphasia care indicator) is summarised in a single measure as the proportion of all recommended processes of care received from the total of applicable processes for a patient. The clinical indicators included in the composite measure were developed using a multistakeholder consensus process comprising a two-round e-Delphi exercise and consensus meetings.^41^

**Table 2:** Primary outcome measure: composite measure of quality indicators received

1	Information about *aphasia* is provided to the person with aphasia.
2	Information about *aphasia* is provided to the person with aphasia’s primary carer(s) and/or communication partner(s).
3	Goal setting is undertaken in partnership with the person with aphasia and their primary carer(s) and communication partner(s).
4	The person with aphasia receives comprehensive aphasia therapy (ie, therapy using a range of formats and treatment approaches, including group and individual therapy, and targeting impairment, activity and participation levels of language and communication).
5	The person with aphasia receives high-dose aphasia therapy (ie, >30 hours of aphasia therapy^20^).

**Table 3:** Study implementation and effectiveness outcomes and timings

Measure	Description	Timing
Implementation outcomes and process evaluation measures (as per Proctor *et al* ^61^)		
Composite measure of quality indicators received (Adoption)	*Primary outcome measure. Quantified composite measure of proportion of quality care indicators received. Data triangulated from video/audio recordings of therapy sessions, weekly therapy logs, clinical progress notes and PWA and SP satisfaction and report of receiving each care component.	After 8 weeks of usual care (UC) or Comprehensive, High-dose Aphasia Treatment (CHAT) intervention
Service reach log (Penetration)	No. of CHAT programme enquiries, No. and source of referrals to CHAT. No. of clinicians recruited, clinician demographics, including age, gender, qualification, years of experience, and role/position. No. of people with aphasia and family members/support people recruited, contact details, demographics (including age, gender, occupation and level of education).	Ongoing*
Behavioural determinants survey (Appropriateness, Adoption)	Total and domain scores derived for treating clinicians based on a self-reported survey, recording changes in attitudes, skills, knowledge, and other barriers, and developed using the Theoretical Domains Framework.^53^	Baseline, post-UC/CHAT, follow-up
Semi-structured interviews and/or focus groups (Acceptability, Feasibility, Adoption)	Conducted with a sample of stakeholders from speech pathology, allied health, nursing, and medical professions to explore the CHAT programme’s suitability, usefulness, and feasibility in the clinical context.	Post-UC/CHAT, mid-implementation, follow-up
Satisfaction questionnaire (Acceptability)	To assess clinician satisfaction with the CHAT programme and intention to continue running the programme after study.	At study completion
Mixed methods fidelity evaluation (Fidelity)	Further details to be published separately.	Throughout implementation
Clinical effectiveness outcomes		
Comprehensive Aphasia Test (CAT)	A standardised assessment of language impairment across modalities: comprehension of spoken and written language, repetition, naming, reading and writing. The CAT Modality Mean provides a reliable estimate of the severity of language impairment and is calculated by taking the mean performance across subtest domains.	Baseline, post-UC/CHAT, follow-up
Communicative Effectiveness Index (CETI)	A proxy-rated measure of participants’ functional communication, rated across 16 items representing different everyday communication situations.	Baseline, post-UC/CHAT, follow-up
Communication Confidence Rating Scale for Aphasia (CCRSA)	A patient-reported outcome measure of communication confidence across 10 communication activities.	Baseline, post-UC/CHAT, follow-up
Stroke and Aphasia Quality of Life-39 (SAQOL-39)	A patient-reported outcome measure of health-related quality of life, scored across physical, communication and psycho-social domains.	Baseline, post-UC/CHAT, follow-up
Semi-structured interviews and/or focus groups	Conducted with a sample of people with aphasia post receipt of the CHAT programme to explore experiences of receiving CHAT and satisfaction with the intervention received.	Following participation in the CHAT programme*
Cost-effectiveness outcomes		
Standardised resource utilisation survey	A patient-reported, resource utilisation survey capturing rehabilitation and service usage during intervention periods (UC/CHAT) and follow-up.	Baseline, post-UC/CHAT, follow-up
EQ-5D-5L Health questionnaire	A patient-reported outcome measure of health-related quality of life across five key dimensions: mobility, self-care, usual activities, pain/discomfort and anxiety/depression, for use in economic evaluations.	Baseline, post-UC/CHAT, follow-up

* During period 3 only.

Triangulated data sourcing will allow independent end-point raters to determine whether an indicator has been met. Data sources will include therapy logs, clinical progress notes, and people with aphasia and support person’s report of receiving each care component. A similar composite measure approach has previously been used for assessing the effects of a quality improvement programme of acute stroke care that employed a prospective, controlled, before-and-after study design.^43^ This study design does not allow for participants and assessors to be blinded to group (ie, usual care and CHAT); however, independent endpoint raters, blinded to group, will be trained for the purpose of scoring 100% of the primary outcome measure.

Secondary implementation outcome measures are summarised in table 3 and will include measures related to programme penetration, appropriateness, acceptability, feasibility and fidelity.

### Clinical effectiveness

Given the complex nature of the intervention,^18 44^ multiple secondary outcome measures have been selected to evaluate the clinical effectiveness of CHAT compared with usual care in the different settings, across International Classification of Functioning, Disability and Health^45^ domains (see table 3). Patient-level effectiveness outcomes will examine language impairment (CAT^39^), communication function (Communicative Effectiveness Index (CETI)^46^), communication confidence (Communication Confidence Rating Scale for Aphasia (CCRSA)^47^) and quality of life (Stroke and Aphasia Quality of Life Scale-39 (SAQOL-39)^48^). Clinical effectiveness outcomes will be obtained at baseline, post-usual care or CHAT treatment (2 months) and at follow-up (5 months). Treating and/or independent speech pathologists will conduct the baseline assessment, whereas all post-treatment and follow-up assessments will be conducted by an independent qualified speech pathologist. All assessments will be conducted either at the health service, at the patient’s home or via telerehabilitation, and will be audio and/or video recorded for fidelity checking. Semistructured interviews will also be conducted with people with aphasia at post-therapy, to explore their experience of receiving CHAT and the perceived clinical effectiveness of the programme. The semistructured interviews will be conducted by a member of the research team.

### Sample size

The sample size calculations were conducted based on a secondary outcome measure of clinical effectiveness, due to the lack of existing data for the primary implementation outcome measure. We conducted a Monte Carlo power analysis using data from two sources: the Language Impairment and Functional Therapy trial (ACTRN12613001182785, n=17) and preliminary findings from the implementation of the CHAT programme at the Surgical Treatment and Rehabilitation Service study (ethics approval: HREC/2020/QRBW/50105, n=43). Model parameters were derived from a random intercept model of the effect of treatment on changes in CETI scores, with an estimated treatment effect of 9.08, a random intercept SD of 14.82 and a residual SD of 8.17. To simulate longitudinal data, each participant was assigned three repeated observations. Based on 1000 Monte Carlo iterations, a sample size of 55 participants per group (110 overall) was estimated to yield at least 80% power to detect the specified effect, assuming a two-tailed alpha of 0.05. With a projected attrition of 20%, a total of 138 participants overall (69 per group) is required.

## Data analyses

### Implementation effectiveness

Median regression will be used to compare differences between usual care and CHAT implementation periods for the primary outcome measure, overall composite score: proportion of quality indicators received. The model will be adjusted for patient clustering by service, to protect against residual confounding. Multilevel logistic regression analysis, with levels defined as patients and hospital, to adjust for patient clustering, will be used for dichotomised outcomes such as those relating to individual quality indicators.

Programme reach and quantitative survey data will be analysed using descriptive statistics (eg, m, SD, IQR). For continuous survey data, mean within-group differences will be calculated using paired t-tests and 95% CI. Qualitative data will be analysed using content analysis^49^ and guided by a theoretically grounded approach to analysis of barriers and facilitators to implementation (eg, Theoretical Domains Framework). Comparative analysis will occur between sources (eg, participants, speech pathologists, allied health assistants) to identify common and distinctive themes.

### Clinical effectiveness

Linear mixed modelling will be used to examine whether changes in clinical effectiveness outcomes (ie, CAT, CETI, SAQOL-39, CCRSA), all of which are on interval scale, vary over time as well as across the two groups (usual care and CHAT), after adjusting for the effects of any potential confounders including linear trends over the usual care versus implementation phases. Before modelling the outcomes, baseline characteristics of the two groups will be examined, with any significant differences considered for modelling as covariates to adjust for their possible confounding effects. Collinearity of the covariates will be assessed before including them in the modelling. The results of the linear mixed models will be presented in the form of ORs and their 95% CI. Both intention-to-treat and per-protocol analyses will be conducted. Qualitative interview data will be analysed using thematic analysis.^50^

### Process evaluation

An embedded mixed-method process evaluation, with a focus on resource allocation, feasibility and treatment fidelity, will be conducted alongside the main study to provide a complimentary analysis on the potential influence of study processes on outcomes as well as inform clinical feasibility and future scale-up. Study adaptations (planned or unintentional) will be recorded using the Framework for Reporting Adaptations and Modifications to Evidence-based Implementation Strategies.^51^ A behavioural determinants survey will be used to explore changes in clinicians’ knowledge, skills and beliefs about implementing the CHAT programme. These data will be compared pre-training, post-training and post-implementation to evaluate changes across timepoints. Semistructured interviews and/or focus groups will be conducted with clinicians and stakeholders to explore the CHAT programme’s suitability, usefulness and feasibility in the clinical context. Qualitative data from semistructured interviews and/or focus groups will be digitally recorded, transcribed and imported into NVivo to assist with data analysis.^52^ Barriers and enablers identified in the interviews and focus groups will be mapped to the Theoretical Domains Framework.^53^ Process and outcome data will be triangulated across qualitative and quantitative data sources. Evaluation of treatment fidelity will be informed by a conceptual fidelity framework,^54^ ^55^ with adaptations to programme delivery explored during interviews or focus groups and through collected intervention data (eg, clinical plans, therapy logs, progress notes, withdrawal forms). Further details on the treatment fidelity protocol will be published separately.

### Economic evaluation

Usual care costs and CHAT programme costs will be calculated and compared using a standard reference year and appropriate unit prices applied, as required. A range of data sources will be used (eg, service-level finance reports, clinician/researcher interviews and patient-level data). We will account for the costs of (1) any additional resources and the extra time taken by therapists and others to provide the CHAT programme (including training, direct and indirect contact hours) over and above usual care; (2) costs of services used by patients including other therapies; (3) patient travel time and expenses and (4) other healthcare resources used. Cost-effectiveness analysis will be performed to determine the net difference in $ per unit improvement on relevant communication outcomes. Cost-utility analysis will include use of quality-adjusted life years as an outcome metric. Multivariable probabilistic and sensitivity analyses will be performed to account for variability in point estimates and assess the robustness of incremental cost-effectiveness ratios based on the different assumptions applied. Implementation costs will be recorded with the Costing Implementation Strategies instrument (Cost-IS tool^56^).

### Ethics and dissemination

Ethical approval for this study has been obtained from the Human Research Ethics Committee of the Royal Brisbane and Women’s Hospital (HREC/2021/QRBW/72154) for all sites. Ethics and research governance approval will be sought for any protocol amendments and site investigators will be notified of any changes. Participant data will be de-identified and stored securely at each site. Electronic data will be stored in a password protected, online research database and will be accessible to research investigators. Data will be kept for at least 7 years from the date of publication as per the National Health and Medical Research Council’s guidelines. Serious adverse events will be monitored, with site investigators responsible for reporting any events to the trial coordinator. An independent physician with experience in stroke will be consulted on a case-by-case basis, and at a minimum of once a year to discuss study progress and safety. Study progress and safety will be reported to the Human Research Ethics Committee, annually. Due to the minimal risk of this study, a Data Monitoring Committee was not deemed necessary.

The study design and reporting will be guided by The Template for Intervention Description and Replication checklist^57^ and the Standards for Reporting Implementation Studies.^58^ Study findings will be disseminated at inter/national conferences and in peer-reviewed journal publications.

### Patient and public involvement

The CHAT programme has been iteratively developed over 12 years to integrate the feedback of people with lived experience of aphasia and to incorporate the latest research evidence. An associate investigator with lived experience of aphasia and the CHAT programme contributed to the development of the grant application, including design selection and development of the research questions. As per the implementation strategy, people with aphasia will contribute to the development of training materials for the implementation of CHAT, including the provision of testimonials. It is anticipated that people with aphasia will be involved in the dissemination of study results through joint presentations to support groups, at local, national and international conferences, and in a customisable accessible format on the Aphasia Research Library.^59^ Patient and public involvement will be reported using the People with Aphasia and Other Layperson Involvement framework.^60^

### Study status

Recruitment for the usual care period of the study was undertaken from April 2023 to February 2025. The intervention transition occurred across health services from August 2024 to June 2025. The CHAT implementation phase commenced in April 2025 and is ongoing.

## Supplementary material

10.1136/bmjopen-2026-117575 online supplemental file 1
