# Supplementary material for: Type 3 hybrid effectiveness-implementation study implementing and evaluating the Comprehensive High-dose Aphasia Treatment (CHAT) programme in Australian rehabilitation services: a protocol
Source: BMJ Open. 2026 May 18;16(5):e117575. doi: 10.1136/bmjopen-2026-117575 (PMC13185025; doi:10.1136/bmjopen-2026-117575)
Supplement: online supplemental file 1 [file bmjopen-16-5-s001.doc]

**Supplemental 1.** Example Participant Information and Consent Form.

**Participant Information Sheet/Consent Form**

**Participants with Aphasia**

*[INSERT SITE]*

| **Title** | Implementation of Comprehensive High-dose Aphasia Treatment (CHAT) |
| --- | --- |
| **Short Title** | CHAT Partnership |
| **Project Sponsor** | The University of Queensland |
| **Coordinating Principal Investigator/ Principal Investigator** | Professor David Copland |
| **Associate Investigator(s)**  **Location**  **Site Principal Investigator** | [INSERT INVESTIGATORS]  [INSERT SITE]  [INSERT INVESTIGATOR] |

**Part 1 What does my participation involve?**

**1 Introduction**

You are invited to take part in this project.

This project is called **Implementation of Comprehensive High-dose Aphasia Treatment.**

You have been invited because **you have aphasia**.

This form tells you about the research project.

It explains **what is involved** with taking part.


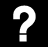


You can ask **questions**.

You can talk with a **family member or friend** about this research.

Participation in this project is **voluntary**. It is **your choice** if you want to participate. You **do not** have to take part in this project.

You will receive **usual speech therapy** if you say **no**.


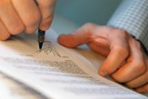
If you want to take part in the project, you will be asked to **sign the consent form**.

By signing you are telling us that you:


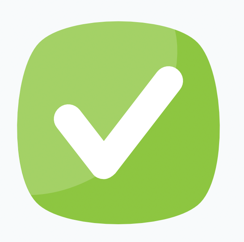


- **Understand** what you have read and been told.
- **Consent to take part** in the research project.
- Consent to the use of your **personal and health information** as described.

You may give us your **verbal or non-verbal consent** if you cannot sign the consent form. Non-verbal consent includes any communication method you usually use that is not talking.

You will be **given a copy** of this Participant Information and Consent Form to keep.

**2 What is the purpose of this research?**

**What is Aphasia?**

Aphasia is a language disorder caused by damage to the brain.

One cause of aphasia is stroke.

Someone with aphasia may have trouble with:


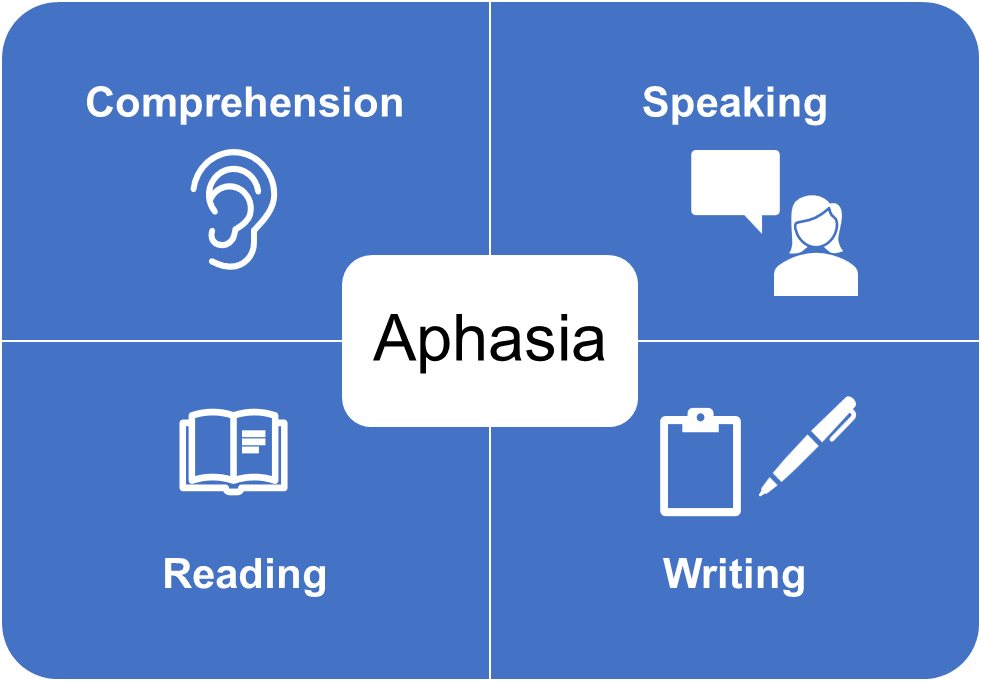


Research tells us that **more aphasia therapy is better** than less aphasia therapy.

**What are the aims of this project?**

We want to **evaluate** the implementation of delivering a comprehensive, high-dose aphasia therapy **(CHAT)** program in the **rehabilitation** setting.

We will:

- **Assess the success** of delivering CHAT in the rehabilitation setting.
- Assess the **cost-effectiveness** of delivering CHAT.
- Investigate if **therapists** report **increased knowledge and skills** in delivering therapy
- Investigate **what affects delivery** of therapy and patient **participation**.

This project will help researchers to deliver **new ways to treat aphasia.**

This research has two parts: (1) **usual care** and (2) **CHAT program**. The usual care phase will run for two years. Then, CHAT therapy phase will run for **the following two years**.

This consent form is for the **CHAT program** phase.

Professor **David Copland** is leading this research.

This research has been **funded** by the **National Health and Medical Research Council (NHMRC).**

This research is being conducted by:

- The University of Queensland
- Southern Cross University
- Monash University
- University of Technology Sydney
- [INSERT SITE]

**3 What does participation in this research involve?**

**What do I need to do?**

**
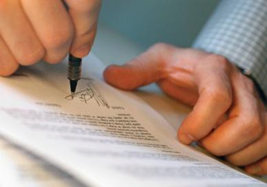
**

Give **permission** to be involved.

Sign a **consent form**. Or, give **verbal or non-verbal consent**.

Start with some **language tests** and **questionnaires** about your communication and quality of life.

Answer **questions** about how your aphasia has affected your life.

Answer questions about **changes to** your **communication** through therapy.

Answer **questions** about your **motivation** for therapy and **how tired** you felt.

Answer questions about **health services** you used during therapy.

Participate in the comprehensive, high-dose **aphasia therapy program (CHAT).**

Therapy is **six (6)** to **seven (7) hours** / **per week** over eight **(8) weeks**.

Do the **language tests** again.


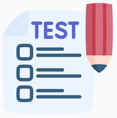

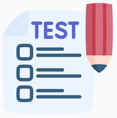

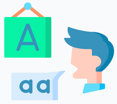


**What will happen?**

**
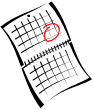
**

You will be involved in the study for about **five (5) months** in total.

**Researchers** will **contact you** before you start assessment.

Assessment

You will complete some **language tests** and **questionnaires.**

You will answer some questions about how your **aphasia has affected** your life.

The tests will take **three (3) to four (4) hours to complete**. You may do the tests over **one (1) or two (2) sessions**.

Therapy

You will set **therapy goals** with your speech pathologist. This will take **1-2 hours**.

You will attend CHAT therapy **six** (6) **to seven** (7) **hours a week** for **eight** (8) **weeks**.

Therapy will include:

- **Impairment** therapy
- Therapy practicing **everyday communication**
- **Computer** therapy
- **Group** therapy
- Family **education**

Therapy may be **face to face** or **online**. Speech pathology **assistants** and / or **student volunteers** may be involved in therapy.

Assessment

After therapy finishes, you will do the **language tests and questionnaires again**. You will answer questions about your aphasia again.

These tests will be repeated again **three (3) months** after therapy ends.

You will do an **interview** about your **views** on the **therapy program**.

Answer questions about your **satisfaction** with the CHAT program.


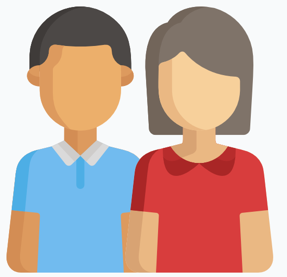


Your **family member or carer** will be asked to **participate** in this study too. They will answer some questions about your aphasia, your therapy and how aphasia affects your life and theirs.

Your **family member or carer** will be invited to **participate** in your **speech** **therapy** sessions.

Your assessment therapy sessions and interviews will be **audio and video recorded**.

Some information will be **obtained from your medical chart (including medical imaging)**. Information will be kept **private**. No identifying details will be given to anyone outside the research team.

**How will this affect me?**

**
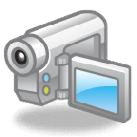
**


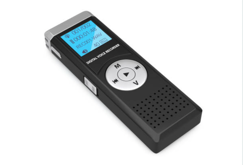
You may be **tape and video recorded**.

There is **no danger** doing this study.

You **may not benefit** from this study.


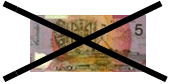
This research will **not cost** you any **money**.

You will **not be paid** for this research.

This project has been designed to make sure the researchers **interpret** the **results fairly** and appropriately. We want to avoid jumping to conclusions.

**4 Other relevant information about the research project**

**
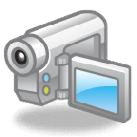

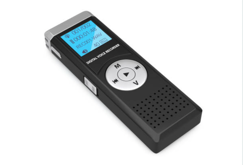
**

If you agree, tape or video **recordings** may be used for **teaching** **and training** purposes.

**Other people with aphasia** will also be doing this study at the same time as you.

We are also **asking staff** and **family members** about their **views** on the **aphasia therapy** program.

**5 Do I have to take part in this research project?**

Your participation is **voluntary**.


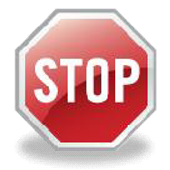


You do **not have to participate**.

You can **stop** at any time.

Saying **no will not affect** your usual **speech therapy**.

Saying no **will not affect** your relationship with:

- The University of Queensland
- Monash University
- Southern Cross University
- University of Technology Sydney
- [INSERT SITE]

**What are the alternatives to participation?**

**
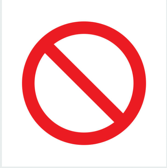
**


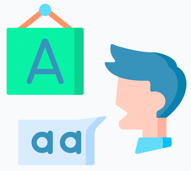

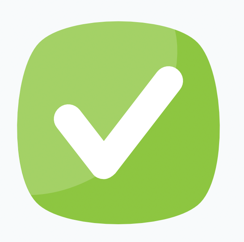
You **can say no** to this research.

You will still get **your usual speech therapy** if you say no.

**7 What are the possible benefits of taking part?**

You **may not benefit** from this study.

This study may help to find **new ways** to **deliver aphasia therapy**.

**8 What are the possible risks and disadvantages of taking part?**

You may experience some **fatigue, stress or frustration** during assessment and therapy sessions**.**


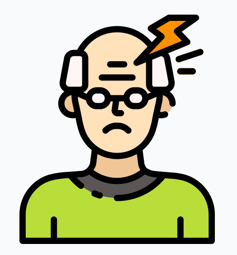
**Tell a researcher** if you feel:

- Tired
- Stressed
- Frustrated

You can **stop** and take a **break**. You can have a **rest**.

**9 What if I withdraw from this research project?**


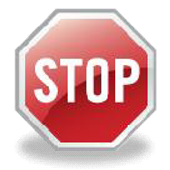
You **do not have to participate** in this study.

You can **stop** at any time.

You can **tell a researcher** if you **want to stop**.

You can **tell a researcher** if you **do not want your information** to be **used** if you withdraw from this study.

**10 What happens when the research project ends?**

**
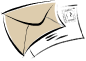
**

You can **ask for the results** of this study.

We can **send the results** to you when we finish the study.

**11 COVID-19 information**

This research follows all **government** and health department **policies** for **COVID-19**. The research follows health orders from:

-
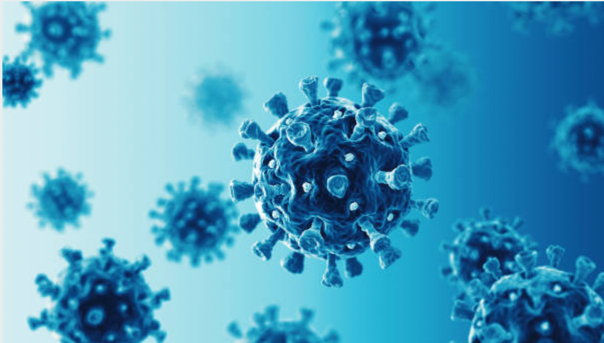
Queensland Government
- New South Wales Government
- South Australian Government
- Federal Australian Government

If your **assessment or** **therapy is interrupted** by COVID-19, you will be provided with options.

1. Complete your assessment and therapy with **telehealth**
2. **Delay** your assessment and therapy until it can be done in-person
3. **Withdraw** from the study

**Part 2 How is the research project being conducted?**

**12 What will happen to information about me?**

**I understand what will happen to information I give:**

**
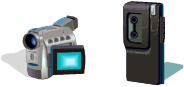
**

I may be **tape / video recorded**.

If I agree, tape or video recordings may be used for **teaching and training** purposes.


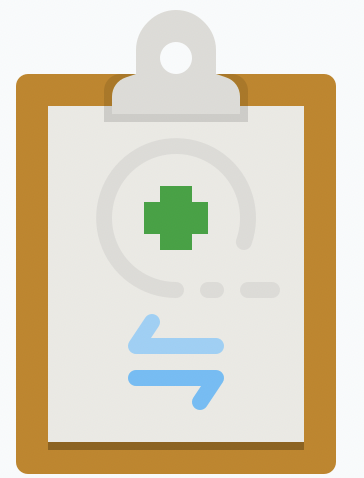


The researchers will look at my **medical chart**.

The researchers will **ask my family** about my **aphasia**.

I will need to give some **personal details**.

For example:

- My **contact details**.
- Information about how I feel about **my aphasia**.

**What will happen to information about me?**

Your information will be **kept private**.

Your information will be kept in a way that we **can identify** you **if needed**.


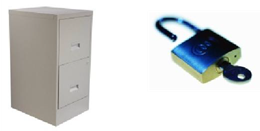


All paper information will be **kept safely** in a locked filing cabinet.

Computer files will be kept on a **password-protected computer**.

The information will be **kept** at **The University of Queensland**.

Only **people who are involved with this study** will look at your **information**. These people will be either researchers, speech pathologists, speech pathology assistants or speech pathology students involved in your therapy.

If I agree, tape or video recordings may be used for **teaching and training** purposes. My identity will be kept **private**.

Your information will be **kept for fifteen (15) years** after the study.


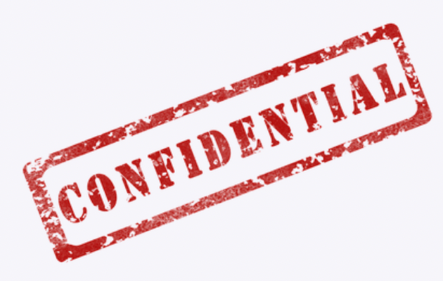
Your information may be used for **future studies**. We will contact you for **consent** before using this information.

All information you give will be **confidential**.

Your **identity** will not be **revealed**.

**Who can check this information?**

Your relevant health records and **information** about you can be **checked** by **The** **University of Queensland**.

This is to check the study procedures and data collected.

By signing the consent form, I allow people to look at this information.

**What will happen with the results?**

The results of this study will be **published in journals**.


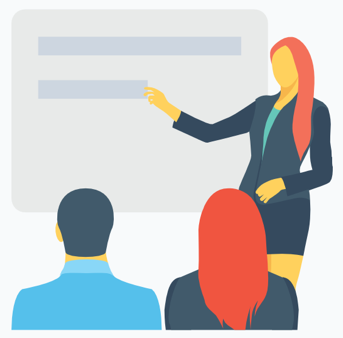


The results of this study will be **presented at conferences**.

My **identity** will be kept **confidential**.

We will give you a **copy of the results** if you would like.

**13 Complaints and compensation**

If you suffer any **harm** in this study **tell the research team**.

You will be given **support** and **treatment**.

**14 Who is organising and funding the research?**

This research project is being conducted by:

- The University of Queensland
- Monash University
- Southern Cross University
- University of Technology Sydney
- [INSERT SITE]

This research is funded by the **National Health and Medical Research Council** (NHMRC).

**
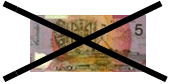
**

The researchers will **not be paid extra money** for you to do this study.

**15 Who has reviewed the research project?**


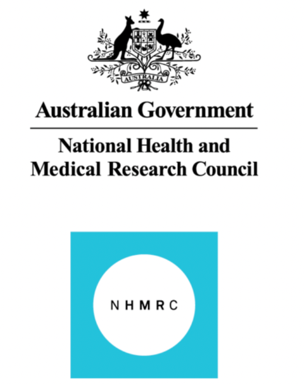
All human research in Australia is reviewed by a **Human Research Ethics Committee** (HREC).

This study has been reviewed by the HRECs of the Royal Brisbane and Women’s Hospital and The University of Queensland.

This project will be carried out according to the *National Statement on Ethical Conduct in Human Research (2007)*.

This statement has been written to **protect people** participating in research.

**16 Further information and who to contact**

**
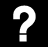
**

If you have **questions** about the **research,** you can contact:

| Name | Research team |
| --- | --- |
| Telephone | INSERT |
| 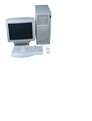Email | INSERT |

OR

| Name | Professor David Copland |
| --- | --- |
| Position | Principal Research Fellow and Speech Pathologist, The University of Queensland |
| Telephone | INSERT |
| 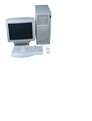Email | INSERT |

If you would like to speak to someone from [INSERT SITE] about the **research** contact:

**Site contact person**

| Name | INSERT SITE CONTACT |
| --- | --- |
| Position | Principal Investigator |
| Telephone | INSERT |
| 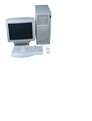Email | INSERT |

Complaints contact person

| Name | Safety, Quality and Innovation Unit |
| --- | --- |
| Position | Patient Liaison Officer |
| Telephone | INSERT |
| 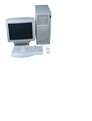Email | INSERT |

**If you want to speak to someone** about:


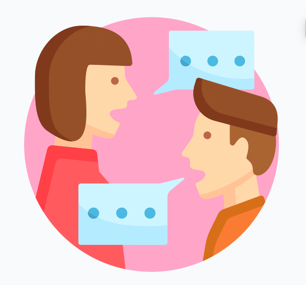


- The way the research is being done.

- Your rights.

- Making a complaint.

You can contact the people below.

These people are not involved in the project.

**Reviewing HREC approving this research and HREC Executive Officer details**

| Reviewing HREC name | INSERT |
| --- | --- |
| Telephone | INSERT |
| 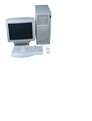Email | INSERT |

**Local RGO Office contact**

| Name | INSERT CONTACT |
| --- | --- |
| Position | Research Governance Officer |
| Telephone | INSERT |
| 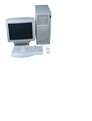Email | INSERT |

**Consent Form**

**Participant with Aphasia**

| **Title** | Implementation of Comprehensive High-dose Aphasia Treatment (CHAT) |
| --- | --- |
| **Short Title** | CHAT Partnership |
| **Project Sponsor** | The University of Queensland |
| **Coordinating Principal Investigator/ Principal Investigator** | Professor David Copland |
| **Associate Investigator(s)**  **Location**  **Site Principal Investigator** | INSERT  INSERT  INSERT |

**Declaration by Participant**

I **understand** the **Participant Information Sheet**.

I **understand** what **this research** is about.

I have **asked questions** and I am happy with the answers.

I **agree to participate** in this project.

I **understand** I can **withdraw** at any time.

If I **stop** this project it will **not affect my care** within [INSERT SITE].

If I **stop** this project it will **not affect my relationships** with The University of Queensland, Monash University, Southern Cross University or University of Technology Sydney.

I understand that I will be given a **signed copy of this form** to keep.

I consent to the use of my de-identified **video and audio** recordings to be used for **teaching and training** purposes

YES  NO 

Name of Participant________________________________________

(please print)

Signature______________________ Date ______________________

OR

Verbal or non-verbal consent obtained (circle): YES / NO

Date: _______

**Declaration by Researcher^†^**

I have given a verbal explanation of the research project; its procedures and risks and I believe that the participant has understood that explanation.

Name of Researcher________________________________________

(please print)

Signature______________________ Date ______________________

^†^ An appropriately qualified member of the research team must provide the explanation of, and information concerning, the research project.

Note: All parties signing the consent section must date their own signature.

**Form for Withdrawal of Participation**

**Participant with Aphasia**

| **Title** | Implementation of Comprehensive High-dose Aphasia Treatment (CHAT) |
| --- | --- |
| **Short Title** | CHAT Partnership |
| **Project Sponsor** | The University of Queensland |
| **Coordinating Principal Investigator/ Principal Investigator** | Professor David Copland |
| **Associate Investigator(s)**  **Location**  **Site Principal Investigator** | INSERT  INSERT  INSERT |

**Declaration by Participant**

I wish to **withdraw from participation** in the above research project.

I understand that this will **not affect my routine care**.

I understand that this will not affect my relationships with the researchers of The University of Queensland, Monash University, Southern Cross University or University of Technology Sydney.

Please select **one** option:

 I **agree** for **my information and data collected** **to be used** by the research team after I withdraw from the study.

 I wish to **withdraw my information and data collected**. I do **not** give permission for this information to be used by the research team.

Name of Participant________________________________________

(please print)

Signature______________________ Date ______________________

OR

Verbal or non-verbal consent obtained (circle): YES / NO

Date: _______

In the event that the participant’s decision to withdraw is communicated verbally, the Senior Researcher must provide a description of the circumstances below.

|  |
| --- |

**Declaration by Researcher^†^**

I have given a verbal explanation of the implications of withdrawal from the research project and I believe that the participant has understood that explanation.

Name of Researcher________________________________________

(please print)

Signature______________________ Date ______________________

^†^ An appropriately qualified member of the research team must provide information concerning withdrawal from the research project.

Note: All parties signing the consent section must date their own signature.
