# Supplementary material for: Type 3 hybrid effectiveness-implementation study implementing and evaluating the Comprehensive High-dose Aphasia Treatment (CHAT) programme in Australian rehabilitation services: a protocol
Source: BMJ Open. 2026 May 18;16(5):e117575. doi: 10.1136/bmjopen-2026-117575 (PMC13185025; doi:10.1136/bmjopen-2026-117575)
Supplement: Supplementary file 2 [file bmjopen-2026-117575.reviewer_comments.pdf]

PEER REVIEW HISTORY

BMJ Open publishes all reviews undertaken for accepted manuscripts. Reviewers are asked to complete a checklist review form and are provided with free text boxes to elaborate on their assessment. These free text comments are reproduced below.

ARTICLE DETAILS

Title (Provisional)

Protocol for a type 3 hybrid effectiveness-implementation study implementing and evaluating the Comprehensive High-dose Aphasia Treatment (CHAT) program in Australian rehabilitation services.

Authors

Mc Sween, Marie-Pier; Dignam, Jade; Shrubsole, Kirstine; Levine, Rachel; Power, Emma; Lannin, Natasha A; Wallace, Sarah J. Jane; Cadilhac, Dominique A; Kilkenny, Monique F; Hill, Annie; Ryan, Brooke; Copland, David A.

VERSION 1 - REVIEW

**Reviewer**	**1**
**Name**	**Doogan, Catherine**
**Affiliation**	**UCL, Translational Neuroscience**
**Date**	**19-Mar-2026**
**COI**	**None**

Thank you for asking me to review this non-randomised pre-post design study with 3 main aims. It is an ambitious and impressive study and I only have some small tweaks for this paper to improve it's readability.

1. It states in the Introduction that CHAT is a modified ICAP (missing one element of an ICAP) but throughout the paper it refers to ICAP's and it is not clear even in the discussion if CHAT is a M-ICAP and if so what element is missing from it. Stating this clearly from the outset would help the reader. Another thing to make clearer earlier is perhaps that the usual care group of participants are not the same as the CHAT intervention participants. What pre-post design is would also benefit from being more clearly defined near the beginning e.g. re-post in each of the 4 stages?

Lastly the role of the Support People and what this is exactly needs to be better explained. Are they merely to be used for CROMS or are they receiving any of the CHAT interventions (e.g. conversation partner training) or does this vary. Also are participants excluded if they do not have a support person?

A very minor point is Figure 1. is quite confusing and I wonder if it could be presented differently? But not necessary.

VERSION 1 - AUTHOR RESPONSE

Reviewer Comment	Response and In-text change, marked version (page, line)
**Reviewer 1**	
It states in the Introduction that CHAT is a modified ICAP (missing one element of an ICAP) but throughout the paper it refers to ICAP's and it is not clear even in the discussion if CHAT is a M-ICAP and if so what element is missing from it. Stating this clearly from the outset would help the reader.	Thank you for raising this issue. CHAT is a modified-ICAP, meeting all but the criteria for treatment intensity, with regards to the defining ICAP elements. This information has now been included in the section "CHAT program intervention" (p. 7). Furthermore, terminology for ICAPs/m-ICAPs has been reviewed for consistency and clarification throughout the manuscript.
Another thing to make clearer earlier is perhaps that the usual care group of participants are not the same as the CHAT intervention participants.	The manuscript has been revised to clarify that the usual care participant group is distinct to the CHAT participant group (Section "Study periods" p. 6-7).
What pre-post design is would also benefit from being more clearly defined near the beginning e.g. re-post in each of the 4 stages?	Clarification of the before-and-after study design has been included in the "Design and setting" section (p. 5) and the study abstract (p. 2).
Lastly the role of the Support People and what this is exactly needs to be better explained. Are they merely to be used for CROMS or are they receiving any of the CHAT interventions (e.g. conversation partner training) or does this vary. Also are participants excluded if they do not have a support person?	Further information about the Support Person's role and involvement in the CHAT program has now been included in the intervention description (p. 7-8).

	"Support people, including family members, significant others and/or carers, are invited to participate in all program elements, as able. This may include attendance at educational group sessions, participation in individual therapy, and technology training and support for computer-based therapy. Depending on the participant with aphasia's therapy goals, support people may also be the direct recipient of intervention (e.g., communication partner training)."<br><br>Participants with aphasia are not required to have a support person consent to be involved in the research. This has been clarified in the section "Participant Recruitment – Support People" (p. 10).
A very minor point is Figure 1. is quite confusing and I wonder if it could be presented differently? But not necessary.	Figure 1 has been revised for clarity.
